# Mcl-1 promotes lung cancer cell migration by directly interacting with VDAC to increase mitochondrial Ca^2+^ uptake and reactive oxygen species generation

**DOI:** 10.1038/cddis.2014.419

**Published:** 2014-10-23

**Authors:** H Huang, K Shah, N A Bradbury, C Li, C White

**Affiliations:** 1 Department of Physiology & Biophysics, Rosalind Franklin University of Medicine & Science, North Chicago, IL, USA; 2 Molecular Targets Program, James Graham Brown Cancer Center, Louisville, KY, USA; 3 Department of Medicine, Pharmacology and Toxicology, University of Louisville, Louisville, KY, USA

## Abstract

Mcl-1 is an antiapoptotic member of the Bcl-2 family frequently upregulated in non-small cell lung carcinoma (NSCLC). We now report the physiological significance of an interaction between Mcl-1 and the mitochondrial outer membrane-localized voltage-dependent anion channel (VDAC) in NSCLC cell lines. Mcl-1 bound with high affinity to VDAC1 and 3 isoforms but only very weakly to VDAC2 and binding was disrupted by peptides based on the VDAC1 sequence. In A549 cells, reducing Mcl-1 expression levels or application of VDAC-based peptides limited Ca^2+^ uptake into the mitochondrial matrix, the consequence of which was to inhibit reactive oxygen species (ROS) generation. In A549, H1299 and H460 cells, both Mcl-1 knockdown and VDAC-based peptides attenuated cell migration without affecting cell proliferation. Migration was rescued in Mcl-1 knockdown cells by experimentally restoring ROS levels, consistent with a model in which ROS production drives increased migration. These data suggest that an interaction between Mcl-1 and VDAC promotes lung cancer cell migration by a mechanism that involves Ca^2+^-dependent ROS production.

The Bcl-2 proteins are a family of molecules comprised of both pro- and antiapoptotic members essential for the regulation of apoptotic cell death. In the classical paradigm, the antiapoptotic proteins Bcl-2, Bcl-x_L_ and Mcl-1, inhibit cell death during receipt of apoptotic stimuli by binding and sequestering the proapoptotic members.^1^ It is now appreciated, however, that in the absence of apoptotic stimuli, Bcl-2 proteins have numerous non-canonical interactions that influence diverse cellular functions, although the precise mechanisms are poorly understood.^2^ Since antiapoptotic Bcl-2 family members are frequently upregulated in cancer, determining if and how these non-canonical interactions confer survival or other advantages to the cancer cell, will be an important step toward identifying new therapeutic targets. One such interaction is with the outer mitochondrial membrane-localized voltage-dependent anion channel (VDAC), a porin channel with three isoforms that serves as a major diffusion pathway for ions and metabolites,^3^ and whose gating properties are affected by either Bcl-2 or Bcl-x_L_ binding.^4, 5, 6^

We recently identified an important role for Bcl-x_L_/VDAC interactions in the regulation of mitochondrial [Ca^2+^].^7^ Moving Ca^2+^ from the cytoplasm to the mitochondrial matrix requires transfer across the outer membrane by VDAC^3,8^ and across the inner membrane by the Ca^2+^ uniporter.^9^ Our studies showed that Bcl-x_L_ interacts with VDAC to facilitate Ca^2+^ uptake into the mitochondrial matrix. It is not known if other Bcl-2 family members, particularly Bcl-2 and Mcl-1, which are also known VDAC binding partners impart the same physiological regulation on mitochondrial [Ca^2+^]. Furthermore, the specific physiological consequences and significance of this regulation remain to be determined.

Increased production and reduced scavenging of reactive oxygen species (ROS) is frequently observed in cancer cells.^10^ While excessive ROS levels are toxic, sub-lethal production serves an important signaling function, particularly in cancers, were ROS promote cell proliferation, migration and invasion.^11, 12, 13, 14, 15^ A primary source of ROS are the mitochondria, and a number of mitochondrial signaling pathways are known to be remodeled and contribute to elevated ROS in cancer cells, including those involved in regulating the electron transport chain (ETC) function and metabolic activity.^11,16, 17, 18^ It is recognized that upregulation of antiapoptotic Bcl-2 proteins are also associated with a pro-oxidant intracellular environment.^19, 20, 21, 22^ Mechanistically, they are thought to act at the level of the mitochondria to affect the respiratory chain and increase production of ROS. Since matrix [Ca^2+^] is an important regulator of mitochondrial metabolism,^23,24^ and as such, contributes to the regulation of mitochondrial ROS production,^25^ we reasoned that antiapoptotic Mcl-1/VDAC interactions could promote ROS generation by facilitating matrix Ca^2+^ uptake.

Understanding non-canonical roles of Mcl-1 is an important step toward identifying novel therapeutic targets, particularly in cancers where it is highly expressed, such as in non-small cell lung cancer (NSCLC).^26,27^ Therefore, we hypothesized that Mcl-1 binding to VDAC promotes mitochondrial Ca^2+^ uptake and ROS production in NSCLC cells and that this is essential in maintaining the cancer cell phenotype. To test this, we assessed the biochemical interaction between Mcl-1 and VDAC and examined the effects of manipulating Mcl-1 expression levels and Mcl-1/VDAC interactions on mitochondrial Ca^2+^ uptake, ROS generation and NSCLC cell proliferation and migration.

## Results

### Mcl-1 binds robustly to VDAC1 and 3 and to VDAC1 with greater apparent affinity than Bcl-x_L_

**Figure 1:**
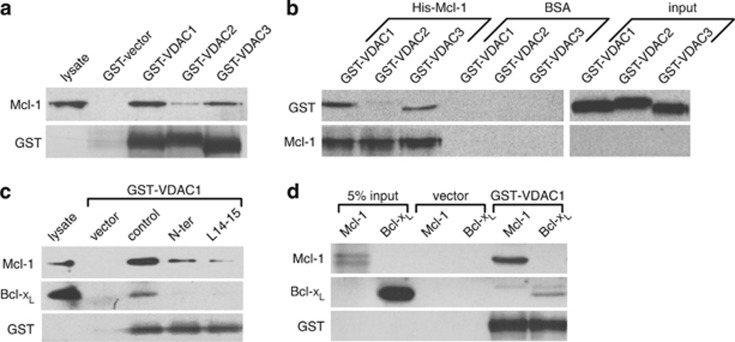
Mcl-1 binds strongly to VDAC1 and 3 and binds to VDAC1 with greater apparent affinity than Bcl-x_L_. (**a**) Human Mcl-1 expressed in MEF cells and detected by western blot after pulldown by GST-fusion proteins of VDAC1, 2 or 3 is shown in the upper lanes with loading control blots of GST depicted below. (**b**) GST-fusion proteins of VDAC1, 2 or 3 detected by anti-GST antibody after pulldown by purified His-tagged Mcl-1 is shown in the upper lanes with the loading control blots of Mcl-1 depicted below. Lanes labeled BSA are negative controls showing GST-VDACs are not pulled down by beads alone. (**c**) Western blot detection of Mcl-1 and Bcl-x_L_ pulled down by GST-VDAC1 from MEF cell lysates pretreated with control or VDAC-based peptides (N-ter and L14-15). (**d**) Western blot of recombinant purified Mcl-1 and Bcl-x_L_ (0.2 *μ*g) pulled down by GST-VDAC1. All blots are representative of at least two independent experiments

To determine the relative binding affinity for the Mcl-1/VDAC interaction, a GST pull-down assay was performed. GST-fusion proteins of VDAC1, 2 and 3 were found to effectively pull down Mcl-1 from lysates of mouse embryonic fibroblasts (MEF) overexpressing human Mcl-1. Similar to the findings reported previously for Bcl-x_L_,^7^ Mcl-1 bound more strongly to VDAC1 compared with VDAC3, and only weakly bound to VDAC2 (Figure 1a). This interpretation was confirmed by the reciprocal experiment in which immobilized His-tagged Mcl-1 robustly pulled down GST-tagged VDAC1 and 3 but not VDAC2 (Figure 1b). Several peptides based on the human VDAC1 sequence (VDAC-based peptides) were tested for their ability to inhibit binding between Mcl-1 and VDAC1. Inclusion of either VDAC-based peptide (N-ter or L14-15) in the reaction mixture effectively reduced both Mcl-1 and Bcl-x_L_ pulldown by GST-VDAC1 (Figure 1c). Intriguingly, compared with the amounts of Mcl-1 and Bcl-x_L_ detected in the input lysate (lane 1, Figure 1c), VDAC1 appeared to pull down a greater proportion of the available Mcl-1 relative to Bcl-x_L_, suggesting that VDAC1 favors binding to Mcl-1 over Bcl-x_L_. This was verified using a GST-VDAC1 pull-down assay baited with equal amounts of purified recombinant Mcl-1 and Bcl-x_L_ (200 ng). Under these conditions a greater proportion of the available Mcl-1 was pulled down by GST-VDAC1, as indicated by comparing the band density of the pulldown to input (Figure 1d; lane 5 and 1 for Mcl-1 and lane 6 and 2 for Bcl-xL). These data confirm that Mcl-1 binds to all three VDAC isoforms, however, the interaction appears strongest with VDAC1 followed by VDAC3 and then only very weakly to VDAC2. Importantly, Mcl-1 binds VDAC1 with a much higher apparent affinity than Bcl-x_L_, suggesting that the Mcl-1/VDAC interaction could have important functional implications.

### Mcl-1 and VDAC interaction increases [Ca^2+^]_mito_ uptake in A549 cells

**Figure 2:**
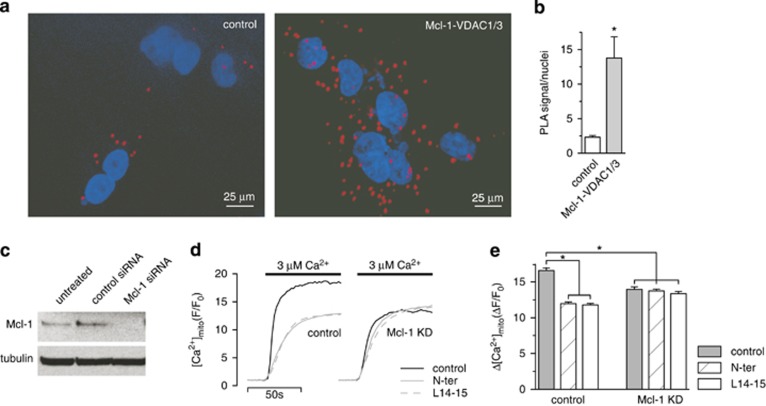
Mcl-1 and VDAC1/3 interact *in vivo* to increase [Ca^2+^]_mito_ uptake. (**a**) An *in situ* proximity ligation assay (PLA) showing the interaction between MCL-1 and VDAC1 and 3 (red) in A549 cells counterstained with DAPI (blue). (**b**) Quantification (mean±S.E.) of the PLA signal normalized to cell number (*P<0.01, student's *t*-test). (**c**) Western blot of A549 cells transfected with control or Mcl-1 siRNA showing efficient knockdown of Mcl-1. Depicted are representative of blots from two independent samples. (**d**, **e**) Shown in **d** are representative traces depicting [Ca^2+^]_mito_ in A549 cells treated with control siRNA or Mcl-1 siRNAs and exposed to step increases in external [Ca^2+^] from 0–3 *μ*M either in the absence or presence of VDAC-based peptides (N-ter or L14-15; 2 *μ*M). The peak [Ca^2+^]_mito_ amplitude (mean±S.E.) of the responses measured in ≥200 cells pooled from two independent samples run on two different days is shown in **e** (*P<0.05, ANOVA)

The A549 cell line is a widely used NSCLC defined by high expression of Mcl-1.^26^ We first confirmed that native Mcl-1 and VDAC1/3 interact in this cell line using a proximity ligation assay (PLA) to detect and quantify the endogenous protein complexes at the single cell level.^28^ The Mcl-1/VDAC complexes were labeled using an antibody directed against Mcl-1 and an antibody known to react with both the VDAC1 and 3 isoforms. Since A549 cells do not express CFTR the assay was also performed using anti-Mcl-1 and anti-CFTR antibodies to control for background signal. Robust PLA signal was observed in the presence of Mcl-1 and VDAC1/3 antibodies compared with control (Figures 2a and b), indicating the presence of Mcl-1/VDAC complexes *in vivo*.

Since we previously demonstrated that regulation of mitochondrial Ca^2+^ ([Ca^2+^]_mito_) uptake was an important functional consequence of the Bcl-x_L_/VDAC interaction,^7^ we asked if the Mcl-1/VDAC interaction similarly affected mitochondrial Ca^2+^ handling. This was first assessed by examining the effect of Mcl-1 knockdown on [Ca^2+^]_mito_ uptake. The effectiveness of siRNA treatment was confirmed by western blot (Figure 2c). Control and Mcl-1 knockdown cells were loaded with the Ca^2+^ indicator Rhod-2 AM and permeabilized with digitonin to release cytoplasmic dye and enable application of solutions directly to the mitochondria. Cells were then bathed in Ca^2+^-free intracellular like media (ICM) containing mitochondrial substrates, and after equilibration, the bathing solution was rapidly switched from 0 Ca^2+^-containing medium to one containing 3 *μ*M [Ca^2+^], and the resultant [Ca^2+^]_mito_ uptake monitored. Compared with control, the magnitude of [Ca^2+^]_mito_ uptake was markedly smaller in Mcl-1 knockdown cells (Figures 2d and e). The small molecule Bcl-2 inhibitor, TW-37, has been shown to effectively block antiapoptotic functions of Mcl-1 mediated through classical BH3-domain-dependent interactions.^29^ The effect of TW-37 on [Ca^2+^]_mito_ uptake was assessed to determine its ability to disrupt the interaction between Mcl-1 and VDAC. The [Ca^2+^]_mito_ uptake measured in response to switching [Ca^2+^] from 0 to 3 *μ*M was 15.4±0.3 (Δ*F*/*F*_0_) under control conditions and was not significantly different (15.6±0.3; *P*>0.05, student's *t*-test) in the presence of 1 *μ*M TW-37 (not shown). This would suggest that the reported physiological effects of TW-37 in regulating cell death^29^ are independent of Mcl-1/VDAC interactions. In contrast, the presence of either VDAC-based peptides (N-ter or L14-15) inhibited [Ca^2+^]_mito_ uptake in control cells but were without effect on Mcl-1 knockdown cells (Figures 2d and e). Taken together, these data indicate that Mcl-1 and VDAC interact *in vivo* to promote Ca^2+^]_mito_ uptake, and may be independent of classical BH3-mediated interactions.

### Mcl-1 promotes mitochondrial ROS production in cancer cells by increasing [Ca^2+^]_mito_ uptake

**Figure 3:**
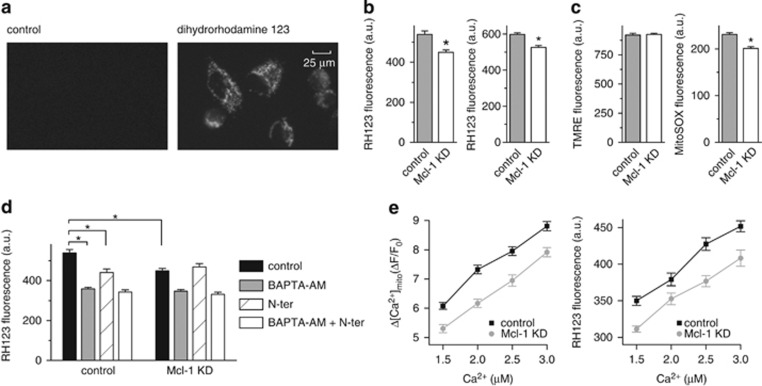
Mcl-1 increases reactive oxygen species (ROS) production in A549 cancer cells by increasing [Ca^2+^]_mito_. (**a**) Representative images showing fluorescence signal from A549 cells before and after loading with the ROS indicator dihydrorhodamine 123. (**b**) Summary bar graphs showing the mean±S.E. (300–400 cells, pooled from three independent experiments; *P<0.05, student's *t*-test) fluorescence signal arising from rhodamine 123 (RH123), the oxidation product of dihydrorhodamine, in A549 cells transfected with control and Mcl-1 siRNA and imaged after equilibration in normal external solution (left bar graph), or after equilibration with 0.5 mM CDNB (right bar graph). (**c**) Summary data for fluorescence imaging performed in control and Mcl-1 siRNA-treated A549 cells after equilibration in normal external solution. Bar graphs depict the mean±S.E. (300–400 cells, pooled from three independent experiments; *P<0.05, student's *t*-test) fluorescence signal from A549 cells loaded with the mitochondrial membrane potential indicator TMRE (left) and the mitochondrial ROS indicator MitoSox (right). (**d**) Summary bar graphs showing RH123 fluorescence recorded in control or Mcl-1 siRNA-treated A549 cells in the absence and presence of VDAC-based peptide (N-ter; 2 *μ*M) alone, BAPTA-AM (25 *μ*M) alone or N-ter and BAPTA-AM in combination. Data represent mean±S.E. of ≥300 cells pooled from three independent experiments (*, *P*<0.05; ANOVA). (**e**) Amplitude of [Ca^2+^]_mito_ uptake (left panel) and RH123 fluorescence (right panel) recorded in permeabilized control and Mcl-1 siRNA-treated A549 in response to switching bathing [Ca^2+^] from 0 to varying [Ca^2+^] in the range 1.5–3 *μ*M. Data represent mean±S.E. of ≥200 cells pooled from four independent experiments

Increased [Ca^2+^]_mito_ is associated with increased mitochondrial ROS production.^25^ To assess the effect of the Mcl-1/VDAC interaction on ROS production, A549 cells were loaded with the ROS indicator, dihydrorhodamine 123 (DHR 123), which concentrates in the mitochondria where it is converted to fluorescent rhodamine 123 (RH123) upon oxidation (Figure 3a). Single cell imaging was performed on control and Mcl-1 siRNA-treated cells. The mean fluorescence intensity of RH123 was lower in Mcl-1 knockdown cells compared with control (Figure 3b), suggesting that Mcl-1 expression is associated with a higher mitochondrial ROS production. It is unlikely that Mcl-1 simply decreases ROS scavenging because in the presence of 1-chloro-2,4-dinitrobenzene (CDNB), a reagent known to inhibit ROS scavenging by depleting glutathione, the RH123 signal was increased in both control and Mcl-1 knockdown cells, but still remained significantly higher in cells expressing Mcl-1 (Figure 3b). Membrane potential was then monitored with the fluorescent indicator tetramethylrhodamine (TMRE). This was done to ensure that differences in RH123 fluorescence were specifically due to differences in ROS and not due to artifacts introduced by changes in membrane potential upon Mcl-1 knockdown. Interestingly, a recent study using a knockout MEF model reported a slight decrease in membrane potential with loss of Mcl-1 measured using a similar approach.^30^ In A549 cells, however, Mcl-1 knockdown using siRNA was without effect on membrane potential (Figure 3c), suggesting that either complete ablation is required or the effect of Mcl-1 on membrane potential is cell-type dependent. To further confirm that Mcl-1 specifically affects mitochondrial ROS, MitoSOX was employed as an alternative mitochondrial redox probe. In MitoSOX-loaded cells, the difference in ROS levels between control and Mcl-1 knockdown was found to be qualitatively similar to that measured with RH123 (Figure 3c).

We next assessed the [Ca^2+^]_mito_-dependence of ROS production. First, intact A549 control and Mcl-1 knockdown cells were incubated with BAPTA-AM, to chelate intracellular Ca^2+^ and block cytoplasmic [Ca^2+^] signals and [Ca^2+^]_mito_ uptake.^7^ BAPTA-AM reduced ROS levels in both control and Mcl-1 knockdown cells to the same level (Figure 3d). In contrast, the VDAC-based peptide (N-ter) which inhibits [Ca^2+^]_mito_ specifically by disrupting the Mcl-1/VDAC interaction (Figures 1 and 2), was only able to reduce ROS levels in control but not in Mcl-1 knockdown cells (Figure 3d). Next, the goal was to monitor the ROS output in control and Mcl-1 knockdown cells under conditions where both cell types experienced the same [Ca^2+^]_mito_. Although the presence of Mcl-1 increases [Ca^2+^]_mito_ uptake, we reasoned that by titrating the [Ca^2+^] in the bathing media, it would be possible to define specific concentrations, that when applied, would evoke similar [Ca^2+^]_mito_ uptake in control and Mcl-1 KD cells. Permeabilized control and Mcl-1 knockdown cells were exposed to 1.5, 2.0, 2.5 or 3.0 *μ*M external Ca^2+^ and the change in [Ca^2+^]_mito_ uptake (Δ*F*/*F*_0_) was measured (Figure 3e; left panel). In separate experiments, ROS levels were monitored in cells similarly challenged with the same [Ca^2+^]-containing solutions (Figure 3e; right panel). These data suggest that ROS levels correlate closely with [Ca^2+^]_mito_ regardless of the expression of Mcl-1. For example, the [Ca^2+^]_mito_ uptake and ROS production in response to the addition of 1.5 *μ*M Ca^2+^ in control cells produced the same [Ca^2+^]_mito_ uptake and ROS production as 2 *μ*M Ca^2+^ in Mcl-1 knockdown cells (Figure 3e). These data are significant because they indicate the importance of mitochondrial Ca^2+^ handling over the transport of other ions or solutes as a primary physiological consequence of the Mcl-1/VDAC interaction.

### Mcl-1 promotes cell migration but not proliferation in NSCLC cells

**Figure 4:**
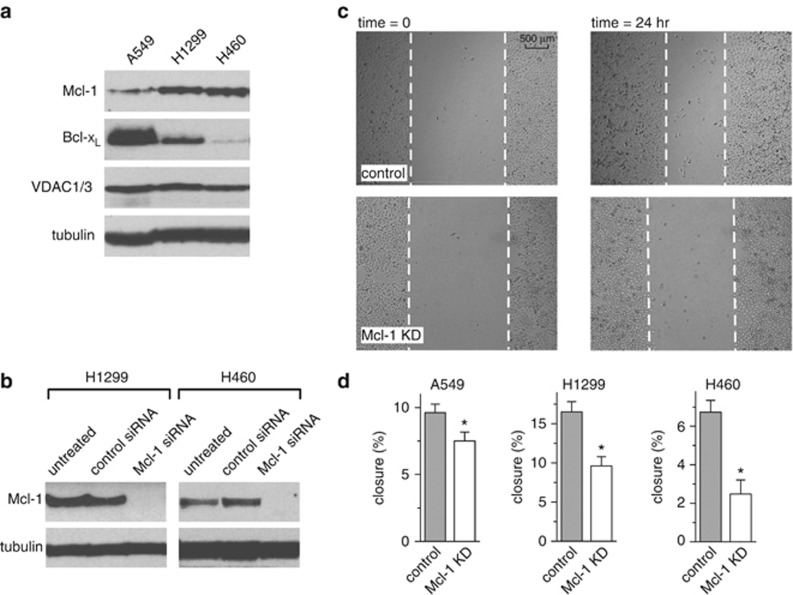
Mcl-1 promotes cell migration in lung cancer cells. (**a**) Western blot showing different expression levels of Mcl-1 and Bcl-x_L_ in three different NSCLC cell lines. (**b**) Western blot of H1299 and H460 cells transfected with control or Mcl-1 siRNA showing efficient knockdown of Mcl-1 in both cell lines. (**c**) Representative images of wound-healing assay showing control and Mcl-1 knockdown A549 cells at 0  and 24 h post scratch. (**d**) Summary bar graphs of the percentage closure in 24 h of control and Mcl-1 knockdown A549, H1299 and H460 cells (mean±S.E.; *, *P*<0.05; student's *t*-test)

**Figure 5:**
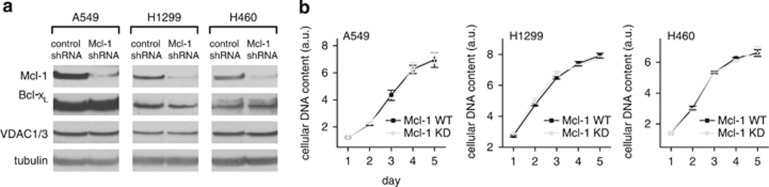
Mcl-1 does not affect cell proliferation in lung cancer cells. (**a**) Western blot showing efficient knockdown of Mcl-1 by shRNA-transfected stable cell lines from A549, H1299 and H460 cells. Bcl-x_L_ and VDAC levels are unaffected. (**b**) Summary of proliferation in control Mcl-1 WT and Mcl-1 knockdown lung cancer cells (mean±S.E.; *P*>0.05; student's *t*-test)

In addition to Mcl-1, both Bcl-2 and Bcl-x_L_ are known to interact with VDAC, raising the possibility of some functional redundancy among the Bcl-2 proteins. Given the difference in apparent binding affinity (Figure 1), however, we hypothesized that in cancer cells overexpressing Bcl-2 proteins, especially those addicted to Mcl-1, Mcl-1/VDAC interactions would dominate. To help define the phenotypes specifically dependent on a Mcl-1/VDAC interaction, we selected three different NSCLC lines (A549, H1299 and H460) previously demonstrated to be similar, with respect to having high Mcl-1 expression,^26,27^ but different in their relative levels of Bcl-x_L_ (Figure 4a). Mitochondrially generated ROS have been defined as a physiological signal that promotes cancer cell migration and proliferation.^12,31, 32, 33^ We employed a scratch wound-healing assay to examine the dependence of Mcl-1 expression in NSCLC cells on these processes. The NSCLC cell lines A549, H1299 and H460 were treated with either control or Mcl-1 siRNAs and the knockdown effectiveness was confirmed by western blot (Figure 4b; see Figure 2c for A549 western blot). The wound area was measured at 0 and 24 h, as depicted for A549 (Figure 4c). In all three cell lines the loss of Mcl-1 expression significantly slowed the rate of wound closure (Figure 4d). This assay, however, does not effectively distinguish between differences in proliferation and migration; therefore, the effect of Mcl-1 knockdown on proliferation was assessed in the three NSCLC cell lines using a microplate assay based on measurement of cellular DNA content. The need to track proliferation across several days in control and Mcl-1 knockdown cells necessitated the generation of stable Mcl-1 knockdowns using shRNAs and antibiotic selection. Western blots confirmed that Mcl-1 was successfully knocked down in A549, H1299 and H460 cell lines without affecting either VDAC1/3 or Bcl-x_L_ expression levels (Figure 5a). Mcl-1 knockdown had no significant effect on cell proliferation (Figure 5b), suggesting that Mcl-1 promotes migration but not proliferation in NSCLC cell lines.

### Increased ROS production by Mcl-1/VDAC interactions promotes NSCLC cell migration

**Figure 6:**
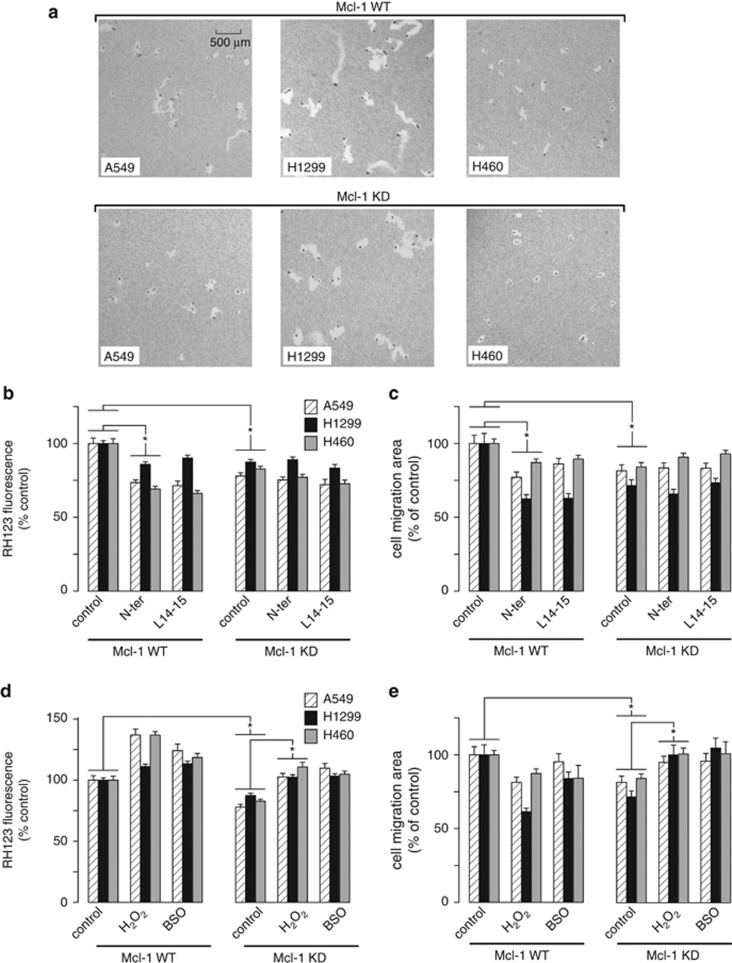
Mcl-1 promotes cell migration by a ROS-dependent mechanism. (**a**) Representative images of the migratory tracks created by both WT and Mcl-1 knockdown A549, H1299 and H460 cells on colloidal gold-coated coverslips in a 24-h time period. (**b**, **c**) The effect of Mcl-1 knockdown and Mcl-1/VDAC inhibition on ROS levels and migration. Bar graphs showing the effect of VDAC-based peptides (N-ter and L14-15; 2 *μ*M) on ROS levels assessed by RH123 (**b**), and cell migration assessed by the migratory track area (**c**) on the cell lines indicated. Data are presented as mean±S.E. normalized to values obtained under control conditions, and represent 100–200 cells pooled from multiple coverslips run on at least two independent days (*P*<0.05; ANOVA). (**d**, **e**) The effect of Mcl-1 knockdown and pharmacological manipulation of steady-state ROS levels on migration. Bar graphs showing the effect of H_2_O_2_ (100 *μ*M) and buthionine sulfoximine (BSO, 100 *μ*M) treatment on ROS levels assessed by RH123 (**d**), and cell migration assessed by the migratory track area (**e**) on the cell lines indicated. Data are presented as mean±S.E. normalized to values obtained under control conditions, and represent 100–200 cells pooled from multiple coverslips run on at least two independent days (*P*<0.05; ANOVA)

We hypothesized that increased ROS production, mediated specifically through the regulation of [Ca^2+^]_mito_ uptake by Mcl-1/VDAC interactions, was necessary and sufficient for Mcl-1 to promote cell migration. To test this, the movement of individual cells was monitored over time by visualizing the tracks left by cells migrating on a colloidal gold-coated surface. Control and stable Mcl-1 knockdown NSCLC cell lines were treated for 24 h under the various conditions described below and the track area created by moving cells was measured as an index of motility (Figure 6a). First, the effect of disrupting the Mcl-1/VDAC interaction using the VDAC-based peptides (N-ter and L14-15) was assessed. As summarized in Figure 6b, application of N-ter and L14-15 significantly reduced ROS production in control A549, H1299 and H460 cells but had no effect after Mcl-1 knockdown. Similarly, N-ter and L14-15 significantly reduced migration in control A549, H1299 and H460 cells but had no effect after Mcl-1 knockdown (Figure 6c). These data demonstrate that both expression of Mcl-1 and its interaction with VDAC are required for Mcl-1 to increase ROS and promote cell migration. Next, we examined the effect of directly manipulating ROS levels on migration by treating NSCLC cells with either H_2_O_2_ (100 *μ*M) or buthionine sulfoximine (100 *μ*M), a glutathione synthesis inhibitor. These interventions increased ROS levels in both control and Mcl-1 knockdown cells, as expected (Figure 6d). Importantly, elevating ROS levels in the Mcl-1 knockdown cells restored migration to levels similar to those observed in control cells (Figure 6e).

## Discussion

A direct interaction between VDAC and antiapoptotic Bcl-2 and Bcl-x_L_ is well-established.^34, 35, 36^ Both Bcl-2 and Bcl-x_L_ bind to the N terminus of VDAC1,^5,36,37^ and as demonstrated using Bcl-x_L_, requires contact with a highly conserved region spanning alpha helices 5 and 6 of the Bcl-2 protein.^38^ The involvement of the BH4 domain of Bcl-2 members has also been implicated,^39,40^ however, this region may be more important in mediating the functional effects rather than contributing to robust binding.^38,39^ We recently demonstrated that in addition to VDAC1, Bcl-x_L_ also binds well to VDAC3 but only very weakly to VDAC2.^7^ To our knowledge there has been only one previous report of an interaction between Mcl-1 and VDAC.^41^ In that study, however, the authors reported that only an N-terminally-truncated fragment of Mcl-1 bound to VDAC1.^41^ This is in contrast to the current study in which immobilized VDAC1 and 3 is readily pulled down by full-length Mcl-1 from cell lysates (Figure 1a). In addition, His-tagged Mcl-1 interacts robustly with recombinant VDAC1 and 3, suggesting direct protein–protein interaction (Figure 1b). This is further supported by the observation that peptides based on the VDAC sequence (N-ter and L14-15) are able to disrupt the interaction (Figure 1c). The VDAC-based peptides used in the current study were originally designed based on the binding determinants of the VDAC1/Bcl-2 interaction,^35^ and later identified as being effective at disrupting VDAC/Bcl-x_L_.^5,7^ The ability of VDAC-based peptides to function as general inhibitors of antiapoptotic Bcl-2 protein-VDAC interactions suggests that the structural determinants governing VDAC binding to Bcl-2, Bcl-x_L_ and Mcl-1 are highly conserved. That being said, we did discover that individual Bcl-2 proteins differ with respect to their relative binding affinity to VDAC. Although binding of Bcl-2 itself was not examined in the current study, we show that Mcl-1 has a much higher apparent affinity for VDAC compared with Bcl-x_L_ (Figures 1c and d). It is likely then that the relative binding affinity in addition to the expression level of each Bcl-2 member governs the structural architecture, and presumably the functional outcome, of the VDAC–Bcl-2 protein complex.

We previously determined that [Ca^2+^]_mito_ uptake was tightly regulated by Bcl-x_L_ through direct interaction with both VDAC1 and VDAC3.^7^ We now show that the VDAC/Mcl-1 interaction confers similar regulation (Figure 2), however, we did not investigate the relative importance of specific VDAC isoforms, choosing instead to focus on defining the functional consequences of enhanced [Ca^2+^]_mito_ uptake. Nevertheless, we do show that Mcl-1 and Bcl-x_L_ bind similarly, in that they both interact more strongly with VDAC1 and VDAC3 compared with VDAC2. The observation that Mcl-1 binds more avidly to VDAC suggests that in cancer cells characterized by Mcl-1 overexpression, the Mcl-1/VDAC interaction could be a major determinant in remodeling mitochondrial physiology. We now provide evidence for this. In A549 cells, known to have high endogenous levels of Mcl-1,^27^ we show that Mcl-1 is in complex with VDAC1/3 (Figures 2a and b), and that either knockdown of Mcl-1 or Mcl-1/VDAC disruption with inhibitor peptides decreases [Ca^2+^]_mito_ uptake by the same amount (Figures 2d and e). However, A549 cells do express Bcl-x_L_, and the Bcl-x_L_/VDAC interactions, which are also sensitive to inhibitor peptides, should contribute to the regulation of [Ca^2+^]_mito_ uptake, as we described.^7^ In that case we might have expected inhibitor peptides to be more effective in lowering [Ca^2+^]_mito_ uptake than Mcl-1 knockdown alone. However, Mcl-1 knockdown and inhibitor peptides are equally effective (Figure 2e), supporting the interpretation that Mcl-1/VDAC, rather than Bcl-x_L_/VDAC, is the dominant interaction in this cell type.

Mitochondrial ROS generation is largely coupled to respiration and occurs when electrons leak from the ETC at complex I and III and react with O_2_ to produce superoxide.^10,25^ Increased ROS generation has also been observed upon Bcl-2, Bcl-x_L_ or Mcl-1 overexpression and linked to mechanisms that result in increased respiration and complex III modulation.^19,20,42,43^ The current study describes a novel mechanism that involves Mcl-1/VDAC interactions. Our conclusion that ROS production is dependent on the Mcl-1/VDAC interaction is supported by the observation that VDAC-based inhibitor peptides decrease ROS in cells expressing Mcl-1 but are without effect when Mcl-1 is knocked down (Figure 3d). These data rule out the possibility that the effects of Mcl-1 on ROS are mediated by Mcl-1 localized to the mitochondrial matrix or at the inner membrane. This is important in light of recent findings that implicate a role for matrix-localized Mcl-1 in regulating bioenergetics and membrane potential.^30^

Evidence for the Ca^2+^ dependence of Mcl-1/VDAC effects on ROS is provided by the observation that Ca^2+^ chelation by BAPTA-AM has the same effect on ROS regardless of Mcl-1 expression or the application of inhibitor peptides (Figure 3d). Moreover, by carefully controlling [Ca^2+^]_mito_ uptake we demonstrate that ROS production is critically dependent on the Ca^2+^ load but not Mcl-1 expression (Figure 3e). These data effectively eliminate alternative interpretations that include the potential for Mcl-1/VDAC to affect ROS by either controlling their release from mitochondria through VDAC,^44^ regulating mitochondrial substrate uptake^6^ or by direct remodeling of the respiratory chain.^42^ Although not investigated in the current study, precedents exist that link [Ca^2+^]_mito_ and ROS generation. Physiological Ca^2+^ uptake into the mitochondrial matrix promotes activation of TCA cycle and generates ROS as a result of increased flux through the ETC.^25^ In addition, Ca^2+^ can enhance electron leak by indirectly inhibiting the ETC at complex III or IV.^45,46^

ROS promotes cancer cell migration and invasion by impinging on a number of signaling pathways, including actin cytoskeleton, cell adhesion and extracellular matrix degradation.^47^ Using a scratch wound-healing assay (Figure 4) we show that wound closure is inhibited by Mcl-1 silencing in all three NSCLC cell lines (A549, H1299 and H460). Since Mcl-1 has no effect on proliferation in these cells (Figure 5), decreased wound closure is most likely mediated by changes in migration (Figure 6a). Importantly, the effect of Mcl-1 knockdown on migration is recapitulated by VDAC-based peptide exposure; moreover, peptides have no additional effect when applied to Mcl-1-knockdown cells (Figure 6c). These data support a model in which Mcl-1 expression in NSCLC cells promotes migration through Mcl-1/VDAC interactions. The conclusion that this is driven by increased ROS is supported by the observation that decreased migration in Mcl-1 knockdown cells is restored by maneuvers designed to elevate ROS (Figure 6e). The delicate balance between the stimulatory and toxic effects of ROS was also noted when experimentally increasing ROS actually decreased migration in control cells (Figure 6e). This is entirely consistent with previous observations in these cell types, demonstrating that excessive ROS leads to decreased proliferation and cell death.^48^

In conclusion, we describe a novel mechanism governing ROS generation and migration in NSCLC cells. These findings are significant because there is a growing body of evidence that links increased mitochondrial ROS generation to increased migration, invasiveness and metastasis in a variety of cancers,^11,14,18,49^ including NSCLC.^12^ Since Mcl-1 is elevated in about 60% of NSCLCs, at levels greater than that observed in any other cancer types,^50^ our data identify the Mcl-1/VDAC interaction as a possible therapeutic target that could limit the metastatic potential in lung cancer.

## Materials and Methods

### Cell culture

NSCLC cell lines A549, H1299 and H460 were obtained from ATCC (Manassas, VA, USA). Cells were maintained in Dulbecco's Modified Eagle Medium (Corning Life Sciences–Mediatech Inc., Manassas, VA, USA) supplemented with 10% Fetal Bovine Serum (Gemini Bioproducts, West Sacramento, CA, USA), 100 U ml^−1^ penicillin (Mediatech Inc.) and 100 *μ*g ml^−1^ streptomycin (Mediatech Inc.) in an incubator with 95% humidity and 5% CO_2_ at 37 °C.

### Solutions and reagents

ICM contained (mM): KCl (120), NaCl (10), KH_2_PO_4_ (1), HEPES (20), sodium succinate (2), EGTA (1) and KOH (pH 7.1). The free [Ca^2+^] was adjusted to the desired level by varying the ratio of Ca^2+^/HEDTA, calculated using maxchelator (C Patton, Stanford University, CA, USA). Chemical reagents were purchased from Sigma-Aldrich (St Louis, MO, USA), except for DHR 123 (Cayman Chemical, Ann Arbor, MI, USA), CDNB (Alfa Aesar, Ward Hill, MA, USA) and Rhod-2 AM, TMRE and MitoSOX (Life Technologies, Grand Island, NY, USA). Recombinant His-tagged Mcl-1 was purchased from Proteintech Group, Inc. (Chicago, IL, USA). Peptides based on the human VDAC1 sequence were synthesized by Biomatik (Wilmington, DE, USA): control (LVLGYEGWLA), N-terminal (GLGKSARDVFTKGYGFG) and L14-15 (LAWTAGNSNTR). Cell-permeant versions were tagged with antennapedia-homeodomain-derived antennapedia (Antp; RQIKIWFQNRRMKWKK) at the carboxyl-terminal of each peptide.

### GST pull-down assay and western blot

GST–VDAC 1, 2 and 3 fusion proteins were generated as described previously,^7^ bound to Glutathione Sepharose 4B (GE Healthcare, Pittsburgh, PA, USA) and incubated with cell lysates, 0.2 *μ*g recombinant Mcl-1 or recombinant Bcl-x_L_. His-tagged Mcl-1 (2 *μ*g) bound to ProBond nickel-chelating resin (Life Technologies) was used to pull down VDAC by incubating with GST–VDAC 1, 2 and 3 fusion proteins. Western blot was performed using antibodies against Mcl-1 (EMD Millipore Billerica, MA, USA), Bcl-x_L_ (BD Biosciences, San Jose, CA, USA), GST (ViroGen Corporation, Watertown, MA, USA) and VDAC1/3 (Abcam; Cambridge, MA, USA).

### Proximity ligation assay

A proximity ligation assay kit (Olink Bioscience, Uppsala, Sweden) was used to study the *in vivo* interaction between Mcl-1 and VDAC1/3. A549 cells were washed in PBS at room temperature and fixed by incubation in Buffer A (1 mM MgCl_2_, 1 mM EGTA, 100 mM PIPES and 3.7% paraformaldehyde, pH 6.5) and Buffer B (100 mM Na_2_B_4_O_7_, 1 mM MgCl_2_, 3.7% paraformaldehyde, pH 11.0) for 5 and 10 min, respectively. Cells were permeabilized with 0.1% Triton X-100 for 30 min and then incubated for 15 min with 50 mM NH_4_Cl before being blocked for 1 h (10% goat serum, 1% BSA in PBS). The remainder of the protocol was carried out following the manufacturer's instructions using rabbit anti-Mcl-1 (Abcam) and mouse anti-VDAC1/3 (Abcam) antibodies to detect Mcl-1/VDAC interactions and mouse monoclonal anti-CFTR (Ab #596; Dr J. Riordan, University of North Carolina, Chapel Hill, NC, USA) as control. The cellular PLA signal was visualized using the PlanApo 60 × , 1.42 NA oil immersion objective of an Olympus IX71 inverted microscope (Olympus America Inc., Center Valley, PA, USA) coupled to a VT-Infinity 3 confocal system (VisiTech International, Sunderland, UK) and quantified using ImageJ software.^51^

### Mcl-1 overexpression and knockdown

Human Mcl-1 was transiently overexpressed as described previously.^52^ Transient transfection of control or Mcl-1 siRNAs (Santa Cruz, Dallas, TX, USA) was carried out 24 h prior to experimentation using Lipofectamine RNAiMAX (Life Technologies) following the manufacturer's instructions. Stable knockdown was achieved using lentiviral transduction particles carrying control or Mcl-1 shRNAs and puromycin (2 *μ*g/ml) selection. Knockdown was confirmed by western blot.

### Mitochondrial [Ca^2+^]

Cells were cultured on glass coverslips and loaded with 3 *μ*M Rhod-2 AM by incubation at 37 ^o^C for 30 min. Cells were then permeabilized by 3–4 min exposure to digitonin (25 *μ*g/ml) applied in Ca^2+^-free ICM. The permeabilized preparation was then allowed to equilibrate in regular Ca^2+^-free ICM for 15 min prior to experimental recording. Coverslips were mounted in a recording chamber on the stage of an inverted IX71 microscope (Olympus America Inc.) and excited at 548 nm. Emitted fluorescence was filtered at 605 nm and collected using a CCD-based imaging system running SimplePCI software (Hamamatsu Corporation, Sewickley, PA, USA). The chamber was continuously perfused with ICM at room temperature, and a rapid solution changer was used to switch to the Ca^2+^ containing solution bathing the cells under study.

### Mitochondrial ROS and membrane potential

Cells were cultured on black optical-bottom 96-well plates. To monitor ROS, cells were incubated in culture medium with 5 *μ*M DHR 123 for 30 min at 37 ^o^C, or 10 *μ*M MitoSOX for 30 min followed by 1 h wash out at 37 ^o^C. For membrane potential measurements, TMRE (15 nM) was added to the medium for 30 min at 37 ^o^C before measurement. In permeabilized cells, 5 *μ*M DHR 123 was added to Ca^2+^-containing ICM and incubated at 37 ^o^C for 30 min. Images were acquired using wide-field fluorescent microscopy. The emission and excitation wavelengths were: 500/535 nm (DHR 123); 548/588 nm (MitoSOX and TMRE).

### Scratch wound-healing assay

Cells were cultured on 24-well plates and wounds were made by scratching the monolayer with a pipette tip. Images of the wounds were taken using a 4 × objective at 0 and 24 h after scratch and the open wound area was measured as a percentage of the total area by TScratch software developed by the Swiss Federal Institute of Technology, Zürich, Switzerland.^53^

### Proliferation assay

Proliferation was assessed using CyQUANT NF Cell Proliferation Assay Kit (Life Technologies) according to the manufacturer's instructions. Briefly, 5000 cells were plated in each well of a 96-well plate. At 24-h intervals, the medium was removed and 50 *μ*l assay mix was added to each well and incubated at 37 ^o^C for 1 h, and florescence intensity measured using a POLARstar Omega plate reader (BMG Labtech Inc., Cary, NC, USA).

### Single cell track migration assay

Colloidal gold-coated 24-well plates were prepared as described in the study by Nogalski *et al.*^54^ One thousand cells were seeded in each coated plate with cultured medium and treatments and cultured for 24 h. Then images were taken using a 1.25 × objective and areas of each single cell track were measured using WIS-PhagoTracker software developed by the Weizmann Institute of Science, Rehovot, Israel.^55,56^ Only tracks containing one cell were used in the analysis.

### Analysis and statistics

Fluorescence microscopy data were collected using a 20 × objective enabling capture of ~50 cells per image field. For all experiments, multiple fields were acquired from each coverslip or well of 96-well plate, and the data were pooled from three to four independent coverslips or wells acquired on at least two different days from independent cultures. When comparison of different cell lines was required, the cells were cultured at the same density and passaged in parallel, and data were acquired on the same day. All fluorescence intensities were background subtracted. Rhod-2 signal was normalized to the initial fluorescence value *F*_0_ and expressed as *F*/*F*_0_. Data were summarized as mean±S.E. and differences between means assessed using the Student's *t*-test for unpaired comparisons. A one-way ANOVA with Fisher's least significant difference *post-hoc* analysis was used for multiple comparisons. For all tests the differences between means were accepted as statistically significant at the 95% confidence level (*P*<0.05).

## Abbreviations

NCSLC: non-small cell lung carcinoma
ROS: reactive oxygen species
VDAC: voltage-dependent anion channel
PLA: proximity ligation assay
TMRE: tetramethylrhodamine
ETC: electron transport chain

## References

[bib1] 1Youle RJ, Strasser A. The BCL-2 protein family: opposing activities that mediate cell death. Nat Rev Mol Cell Biol 2008; 9: 47–59. 18097445 10.1038/nrm2308

[bib2] 2Hardwick JM, Soane L. Multiple functions of BCL-2 family proteins. Cold Spring Harb Perspect Biol 2013; 5: a008722. 23378584 10.1101/cshperspect.a008722 PMC3552500

[bib3] 3Colombini M. VDAC structure, selectivity, and dynamics. Biochim Biophys Acta 2012; 1818: 1457–1465. 22240010 10.1016/j.bbamem.2011.12.026 PMC3327780

[bib4] 4Shimizu S, Shinohara Y, Tsujimoto Y. Bax and Bcl-xL independently regulate apoptotic changes of yeast mitochondria that require VDAC but not adenine nucleotide translocator. Oncogene 2000; 19: 4309–4318. 10980606 10.1038/sj.onc.1203788

[bib5] 5Arbel N, Ben-Hail D, Shoshan-Barmatz V. Mediation of the anti-apoptotic activity of BCL-XL upon interaction with VDAC1. J Biol Chem 2012; 287: 23152–23161. 22589539 10.1074/jbc.M112.345918 PMC3391160

[bib6] 6Vander Heiden MG, Li XX, Gottleib E, Hill RB, Thompson CB, Colombini M. Bcl-xL promotes the open configuration of the voltage- dependent anion channel and metabolite passage through the outer mitochondrial membrane. J Biol Chem 2001; 276: 19414–19419. 11259441 10.1074/jbc.M101590200

[bib7] 7Huang H, Hu X, Eno CO, Zhao G, Li C, White C. An interaction between Bcl-xL and VDAC promotes mitochondrial Ca^2+^ uptake. J Biol Chem 2013; 288: 19870–19881. 23720737 10.1074/jbc.M112.448290 PMC3707689

[bib8] 8Rapizzi E, Pinton P, Szabadkai G, Wieckowski MR, Vandecasteele G, Baird G et al. Recombinant expression of the voltage-dependent anion channel enhances the transfer of Ca^2+^ microdomains to mitochondria. J Cell Biol 2002; 159: 613–624. 12438411 10.1083/jcb.200205091 PMC2173108

[bib9] 9De Stefani D, Raffaello A, Teardo E, Szabò I, Rizzuto R. A forty-kilodalton protein of the inner membrane is the mitochondrial calcium uniporter. Nature 2011; 476: 336–340. 21685888 10.1038/nature10230 PMC4141877

[bib10] 10Hamanaka RB, Chandel NS. Mitochondrial reactive oxygen species regulate cellular signaling and dictate biological outcomes. Trends Biochem Sci 2010; 35: 505–513. 20430626 10.1016/j.tibs.2010.04.002 PMC2933303

[bib11] 11Pelicano H, Lu W, Zhou Y, Zhang W, Chen Z, Hu Y et al. Mitochondrial dysfunction and reactive oxygen species imbalance promote breast cancer cell motility through a CXCL14-mediated mechanism. Cancer Res 2009; 69: 2375–2383. 19276362 10.1158/0008-5472.CAN-08-3359 PMC2760349

[bib12] 12Luanpitpong S, Talbott SJ, Rojanasakul Y, Nimmannit U, Pongrakhananon V, Wang L et al. Regulation of lung cancer cell migration and invasion by reactive oxygen species and caveolin-1. J Biol Chem 2010; 285: 38832–38840. 20923773 10.1074/jbc.M110.124958 PMC2998081

[bib13] 13Ma J, Zhang Q, Chen S, Fang B, Yang Q, Chen C et al. Mitochondrial dysfunction promotes breast cancer cell migration and invasion through HIF1α accumulation *via* increased production of reactive oxygen species. PLoS One 2013; 8: e69485. 23922721 10.1371/journal.pone.0069485 PMC3726697

[bib14] 14Ishikawa K, Takenaga K, Akimoto M, Koshikawa N, Yamaguchi A, Imanishi H et al. ROS-generating mitochondrial DNA mutations can regulate tumor cell metastasis. Science 2008; 320: 661–664. 18388260 10.1126/science.1156906

[bib15] 15Sharma LK, Fang H, Liu J, Vartak R, Deng J, Bai Y. Mitochondrial respiratory complex I dysfunction promotes tumorigenesis through ROS alteration and AKT activation. Hum Mol Genet 2011; 20: 4605–4616. 21890492 10.1093/hmg/ddr395 PMC3209831

[bib16] 16Weinberg F, Hamanaka R, Wheaton WW, Weinberg S, Joseph J, Lopez M et al. Mitochondrial metabolism and ROS generation are essential for Kras-mediated tumorigenicity. Proc Natl Acad Sci USA 2010; 107: 8788–8793. 20421486 10.1073/pnas.1003428107 PMC2889315

[bib17] 17Anastasiou D, Poulogiannis G, Asara JM, Boxer MB, Jiang J, Shen M et al. Inhibition of pyruvate kinase M2 by reactive oxygen species contributes to cellular antioxidant responses. Science 2011; 334: 1278–1283. 22052977 10.1126/science.1211485 PMC3471535

[bib18] 18Hung W-Y, Huang K-H, Wu C-W, Chi C-W, Kao H-L, Li AF-Y et al. Mitochondrial dysfunction promotes cell migration *via* reactive oxygen species-enhanced β5-integrin expression in human gastric cancer SC-M1 cells. Biochim Biophys Acta 2012; 1820: 1102–1110. 22561002 10.1016/j.bbagen.2012.04.016

[bib19] 19Chen ZX, Pervaiz S. Bcl-2 induces pro-oxidant state by engaging mitochondrial respiration in tumor cells. Cell Death Differ 2007; 14: 1617–1627. 17510660 10.1038/sj.cdd.4402165

[bib20] 20Kowaltowski AJ, Fenton RG, Fiskum G. Bcl-2 family proteins regulate mitochondrial reactive oxygen production and protect against oxidative stress. Free Radic Biol Med 2004; 37: 1845–1853. 15528043 10.1016/j.freeradbiomed.2004.09.005

[bib21] 21Steinman HM. The Bcl-2 oncoprotein functions as a pro-oxidant. J Biol Chem 1995; 270: 3487–3490. 7876080

[bib22] 22Lee M, Hyun DH, Marshall KA, Ellerby LM, Bredesen DE, Jenner P et al. Effect of overexpression of BCL-2 on cellular oxidative damage, nitric oxide production, antioxidant defenses, and the proteasome. Free Radic Biol Med 2001; 31: 1550–1559. 11744329 10.1016/s0891-5849(01)00633-5

[bib23] 23Jouaville LS, Pinton P, Bastianutto C, Rutter GA, Rizzuto R. Regulation of mitochondrial ATP synthesis by calcium: evidence for a long-term metabolic priming. Proc Natl Acad Sci USA 1999; 96: 13807–13812. 10570154 10.1073/pnas.96.24.13807 PMC24146

[bib24] 24Pitter JG, Maechler P, Wollheim CB, Spät A. Mitochondria respond to Ca^2+^ already in the submicromolar range: correlation with redox state. Cell Calcium 2002; 31: 97–104. 11969250 10.1054/ceca.2001.0264

[bib25] 25Brookes PS, Yoon Y, Robotham JL, Anders MW, Sheu S-S. Calcium, ATP, and ROS: a mitochondrial love-hate triangle. Am J Physiol Cell Physiol 2004; 287: C817–C833. 15355853 10.1152/ajpcell.00139.2004

[bib26] 26Zhang H, Guttikonda S, Roberts L, Uziel T, Semizarov D, Elmore SW et al. Mcl-1 is critical for survival in a subgroup of non-small-cell lung cancer cell lines. Oncogene 2011; 30: 1963–1968. 21132008 10.1038/onc.2010.559

[bib27] 27Song L, Coppola D, Livingston S, Cress D, Haura EB. Mcl-1 regulates survival and sensitivity to diverse apoptotic stimuli in human non-small cell lung cancer cells. Cancer Biol Ther 2005; 4: 267–276. 15753661 10.4161/cbt.4.3.1496

[bib28] 28Söderberg O, Gullberg M, Jarvius M, Ridderstråle K, Leuchowius K-J, Jarvius J et al. Direct observation of individual endogenous protein complexes *in situ* by proximity ligation. Nat Methods 2006; 3: 995–1000. 17072308 10.1038/nmeth947

[bib29] 29Varadarajan S, Vogler M, Butterworth M, Dinsdale D, Walensky LD, Cohen GM. Evaluation and critical assessment of putative MCL-1 inhibitors. Cell Death Differ 2013; 20: 1475–1484. 23832116 10.1038/cdd.2013.79 PMC3792441

[bib30] 30Perciavalle RM, Stewart DP, Koss B, Lynch J, Milasta S, Bathina M et al. Anti-apoptotic MCL-1 localizes to the mitochondrial matrix and couples mitochondrial fusion to respiration. Nat Cell Biol 2012; 14: 575–583. 22544066 10.1038/ncb2488 PMC3401947

[bib31] 31Novo E, Marra F, Zamara E, Valfrè di Bonzo L, Caligiuri A, Cannito S et al. Dose dependent and divergent effects of superoxide anion on cell death, proliferation, and migration of activated human hepatic stellate cells. Gut 2006; 55: 90–97. 16041064 10.1136/gut.2005.069633 PMC1856394

[bib32] 32Nishikawa M. Reactive oxygen species in tumor metastasis. Cancer Lett 2008; 266: 53–59. 18362051 10.1016/j.canlet.2008.02.031

[bib33] 33Sanuphan A, Chunhacha P, Pongrakhananon V, Chanvorachote P. Long-term nitric oxide exposure enhances lung cancer cell migration. Biomed Res Int 2013; 2013: 186972. 23984323 10.1155/2013/186972 PMC3747486

[bib34] 34Shimizu S, Narita M, Tsujimoto Y. Bcl-2 family proteins regulate the release of apoptogenic cytochrome c by the mitochondrial channel VDAC. Nature 1999; 399: 483–487. 10365962 10.1038/20959

[bib35] 35Arbel N, Shoshan-Barmatz V. Voltage-dependent anion channel 1-based peptides interact with Bcl-2 to prevent antiapoptotic activity. J Biol Chem 2010; 285: 6053–6062. 20037155 10.1074/jbc.M109.082990 PMC2825399

[bib36] 36Abu-Hamad S, Arbel N, Calo D, Arzoine L, Israelson A, Keinan N et al. The VDAC1 N-terminus is essential both for apoptosis and the protective effect of anti-apoptotic proteins. J Cell Sci 2009; 122: 1906–1916. 19461077 10.1242/jcs.040188

[bib37] 37Geula S, Ben-Hail D, Shoshan-Barmatz V. Structure-based analysis of VDAC1: N-terminus location, translocation, channel gating and association with anti-apoptotic proteins. Biochem J 2012; 444: 475–485. 22397371 10.1042/BJ20112079

[bib38] 38Malia TJ, Wagner. NMR Structural Investigation of the Mitochondrial Outer Membrane Protein VDAC and Its Interaction with Antiapoptotic Bcl-xL†. Biochemistry 2006; 46: 514–525. 10.1021/bi061577h PMC2579276 17209561

[bib39] 39Shimizu S, Konishi A, Kodama T, Tsujimoto Y. BH4 domain of antiapoptotic Bcl-2 family members closes voltage-dependent anion channel and inhibits apoptotic mitochondrial changes and cell death. Proc Natl Acad Sci USA 2000; 97: 3100–3105. 10737788 10.1073/pnas.97.7.3100 PMC16199

[bib40] 40Shi Y, Chen J, Weng C, Chen R, Zheng Y, Chen Q et al. Identification of the protein-protein contact site and interaction mode of human VDAC1 with Bcl-2 family proteins. Biochem Biophys Res Commun 2003; 305: 989–996. 12767928 10.1016/s0006-291x(03)00871-4

[bib41] 41Weng C, Li Y, Xu D, Shi Y, Tang H. Specific cleavage of Mcl-1 by caspase-3 in tumor necrosis factor-related apoptosis-inducing ligand (TRAIL)-induced apoptosis in Jurkat leukemia T cells. J Biol Chem 2005; 280: 10491–10500. 15637055 10.1074/jbc.M412819200

[bib42] 42Chen W-L, Kuo K-T, Chou T-Y, Chen C-L, Wang C-H, Wei Y-H et al. The role of cytochrome c oxidase subunit Va in non-small cell lung carcinoma cells: association with migration, invasion and prediction of distant metastasis. BMC Cancer 2012; 12: 273. 22748147 10.1186/1471-2407-12-273 PMC3406994

[bib43] 43Chen ZX, Pervaiz S. Involvement of cytochrome c oxidase subunits Va and Vb in the regulation of cancer cell metabolism by Bcl-2. Cell Death Differ 2010; 17: 408–420. 19834492 10.1038/cdd.2009.132

[bib44] 44Tikunov A, Johnson CB, Pediaditakis P, Markevich N, Macdonald JM, Lemasters JJ et al. Closure of VDAC causes oxidative stress and accelerates the Ca^2+^-induced mitochondrial permeability transition in rat liver mitochondria. Arch Biochem Biophys 2010; 495: 174–181. 20097153 10.1016/j.abb.2010.01.008 PMC2855314

[bib45] 45Grijalba MT, Vercesi AE, Schreier S. Ca^2+^-induced increased lipid packing and domain formation in submitochondrial particles. A possible early step in the mechanism of Ca2+-stimulated generation of reactive oxygen species by the respiratory chain. Biochemistry 1999; 38: 13279–13287. 10529202 10.1021/bi9828674

[bib46] 46Brookes P, Darley-Usmar VM. Hypothesis: the mitochondrial NO signaling pathway, and the transduction of nitrosative to oxidative cell signals: an alternative function for cytochrome C oxidase. Free Radic Biol Med 2002; 32: 370–374. 11841927 10.1016/s0891-5849(01)00805-x

[bib47] 47Hurd TR, DeGennaro M, Lehmann R. Redox regulation of cell migration and adhesion. Trends Cell Biol 2012; 22: 107–115. 22209517 10.1016/j.tcb.2011.11.002 PMC4515034

[bib48] 48Laurent A, Nicco C, Chéreau C, Goulvestre C, Alexandre J, Alves A et al. Controlling tumor growth by modulating endogenous production of reactive oxygen species. Cancer Res 2005; 65: 948–956. 15705895

[bib49] 49Taddei ML, Giannoni E, Raugei G, Scacco S, Sardanelli AM, Papa S et al. Mitochondrial oxidative stress due to complex i dysfunction promotes fibroblast activation and melanoma cell invasiveness. J Signal Transduct 2012; 2012: 684592. 22272371 10.1155/2012/684592 PMC3261495

[bib50] 50Beroukhim R, Mermel CH, Porter D, Wei G, Raychaudhuri S, Donovan J et al. The landscape of somatic copy-number alteration across human cancers. Nature 2010; 463: 899–905. 20164920 10.1038/nature08822 PMC2826709

[bib51] 51Schneider CA, Rasband WS, Eliceiri KW. NIH Image to ImageJ: 25 years of image analysis. Nat Methods 2012; 9: 671–675. 22930834 10.1038/nmeth.2089 PMC5554542

[bib52] 52Eckenrode EF, Yang J, Velmurugan GV, Foskett JK, White C. Apoptosis protection by Mcl-1 and Bcl-2 modulation of inositol 1,4,5-trisphosphate receptor-dependent Ca^2+^ signaling. J Biol Chem 2010; 285: 13678–13684. 20189983 10.1074/jbc.M109.096040 PMC2859530

[bib53] 53Gebäck T, Schulz MMP, Koumoutsakos P, Detmar M. TScratch: a novel and simple software tool for automated analysis of monolayer wound healing assays. Biotechniques 2009; 46: 265–274. 19450233 10.2144/000113083

[bib54] 54Nogalski MT, Chan GCT, Stevenson EV, Collins-McMillen DK, Yurochko AD. A quantitative evaluation of cell migration by the phagokinetic track motility assay. J Vis Exp 2012; 4: e4165. 10.3791/4165 PMC3567159 23242175

[bib55] 55Naffar-Abu-Amara S, Shay T, Galun M, Cohen N, Isakoff SJ, Kam Z et al. Identification of novel pro-migratory, cancer-associated genes using quantitative, microscopy-based screening. PLoS One 2008; 3: e1457. 18213366 10.1371/journal.pone.0001457 PMC2195451

[bib56] 56Sharon E, Galun M, Sharon D, Basri R, Brandt A. Hierarchy and adaptivity in segmenting visual scenes. Nature 2006; 442: 810–813. 16810176 10.1038/nature04977

